# Efficacy and Satisfaction of Low Doses UVA1 Phototherapy: A Spanish Experience from a Single Centre

**DOI:** 10.3390/life13030669

**Published:** 2023-02-28

**Authors:** Juan Pablo Velasco-Amador, Laura Linares-Gonzalez, Francisco Javier De la Torre-Gomar

**Affiliations:** Department of Dermatology, Hospital Universitario San Cecilio, 18016 Granada, Spain

**Keywords:** UVA1, phototherapy, low dose, satisfaction, atopic dermatitis, cutaneous T cell lymphoma, inflammatory, morphea, keloids, sclerotic, ultraviolet A1

## Abstract

Background: UVA1 phototherapy is a treatment used for multiple dermatological conditions. The optimal therapeutic regimens and dosing of UVA1 are a matter of debate. The dosages used vary widely between published studies and there are no evidence-based protocols that provide data on dosage, duration, or the role of maintenance therapy. The purpose of this study is to evaluate the experience in our medical center regarding treatment with UVA1, as well as the degree of patient satisfaction with the treatment according to their pathology. Methods: We present a retrospective evaluation of outcomes, treatment tolerability, and satisfaction in adult patients using a low dose of UVA1 phototherapy, administered in our dermatologic service between 2019 and 2022. Results: A total of 78 patients were treated with UVA1, of whom 46 patients (59%) were over 18 years old, completed treatment, and gave their consent. The overall objective clinical response rate was 91.30% (42/46), achieving a complete response in 17 (36.96%) patients, partial response in 25 (54.34%), and no response in 4 (8.70%). The complete response rates recorded were high in morphea, scleredema, or chronic hand eczema. In terms of the level of satisfaction objectively measured by TSQM-9 version 1.4, highlighting high scores obtained in mastocytosis, systemic sclerosis, morphea, scleredema, chronic hand eczema, or prurigo nodularis (over 65 points). Conclusions: We present a review of treatment with UVA1 phototherapy at low doses with good response in a wide variety of dermatological pathologies.

## 1. Introduction

Phototherapy is used for the treatment of various dermatological conditions and involves controlled exposure of the skin to predetermined ranges of ultraviolet rays. UV light can be divided into different radiation ranges [1]. UVA1 phototherapy uses the non-erythematogenic wavelength (340–400 nm), reducing the risk of light burns associated with UVA2 (320–340 nm) and UVB (290–320 nm) radiation [1]. UVA1 dosimetry is categorized into low- (10 to 30 J/cm^2^), medium- (31 to 60 J/cm^2^), and high- (>60 J/cm^2^) dose regimens [1]. The cumulative dose per phototherapy cycle is another classification point, with low being less than 300 J/cm^2^, medium being 300–975 J/cm^2^, and high being 975–1840 J/cm^2^ [1]. Low-dose UVA1 is generated by fluorescent lamps, whereas medium and high doses require high-intensity-emitting metal–halide lamps [1]. UVA1 phototherapy is recognized as a treatment option to be taken into account in several sclerotic diseases (morphea, systemic sclerosis, extragenital lichen sclerosus, chronic sclerodermic graft versus host disease) and in several inflammatory dermatoses, such as psoriasis, atopic dermatitis, systemic lupus erythematosus, cutaneous mastocytosis, hypereosinophilic syndrome, granuloma annulare, keloids, idiopathic follicular mucinosis, and cutaneous T-cell lymphoma [1,2]. The treatment will range from 10 min to 1 h per session, with usually between 3 and 5 sessions per week. Its therapeutic capacity resides in its ability to penetrate the dermis deeper than other modalities of phototherapy, such as UVB, targeting cells located in the superficial dermis [2,3,4]. The purpose of this study is to evaluate the experience in our medical center treated with UVA1 assessing the response rate of UVA1 phototherapy used for inflammatory, sclerotic, and neoplastic dermatological diseases, as well as the degree of patient satisfaction with the treatment according to their pathology. We prescribe a low-dose phototherapy regimen based on some studies that support the hypothesis about the importance of the cumulative dose, rather than the use of high individual doses in short periods of treatment, and on the possibilities offered by our equipment [2].

## 2. Materials and Methods

We collected data from the clinical record of our phototherapy unit from 2019 until 2022, recording patient age, gender, diagnosis (confirmed histopathologically where necessary), cumulative treatment dose, number of sessions per treatment cycle, assessment of clinical outcome, and adverse effects in patients over 18 years of age who completed treatment and gave their consent.

Clinical assessments were made before and after UVA1 phototherapy by the same physician. It was based on some of the main scales of measurement for each entity and summarized as complete, partial, or no response compared with the baseline presentation. To objectify the therapeutic response of our patients, we used the modified Rodnan skin score in scleroderma, the Detroit scale in keloids, the body surface area score (BSA) in granuloma annulare, the eczema area and severity index score (EASI) and the investigator’s global assessment scale (IGA) in atopic dermatitis, the hand eczema severity index (HECSI) in chronic hand eczema, the modified severity-weighted assessment tool (mSWAT) in mycosis fungoides, the BSA in mucinosis, the analogic visual scale (EVA) pruritus scale in mastocytosis and prurigo, and the BSA score and modified Rodnan skin score in lichen sclerosus. We assessed patient satisfaction using the treatment satisfaction questionnaire for medication (TSQM-9 version 1.4).

**Figure 1:**
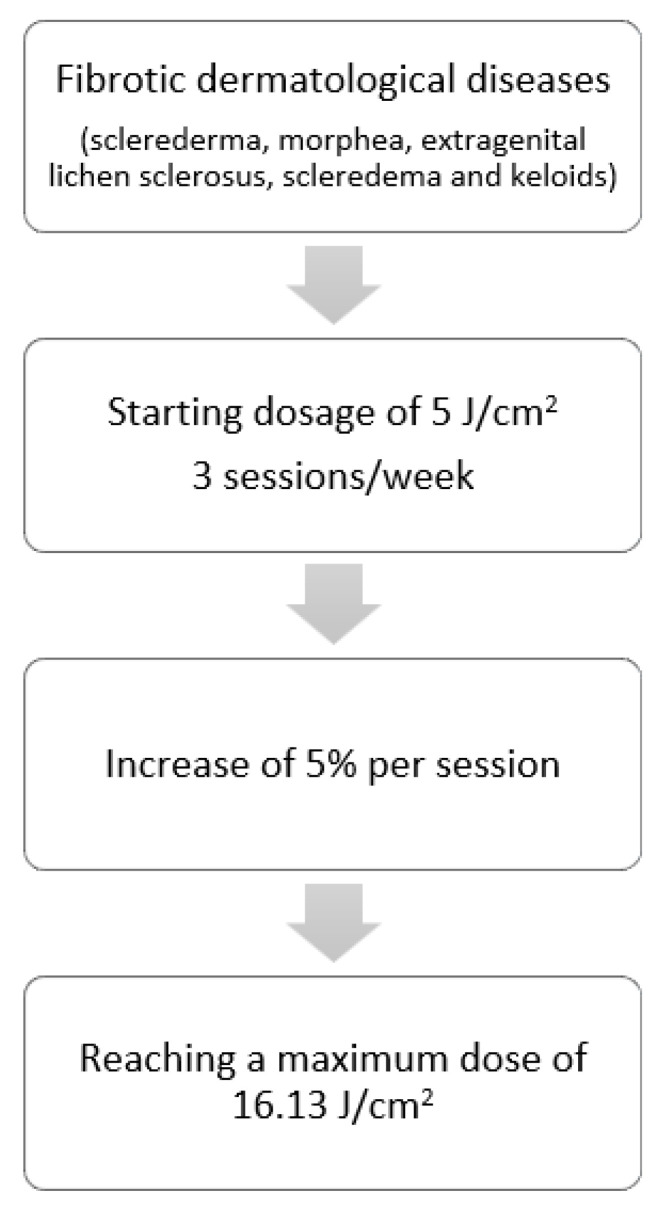
Treatment regimens for fibrotic dermatological diseases.

Patients with the diagnosis of fibrotic skin diseases (sclerederma, morphea, extragenital lichen sclerosus, scleredema, and keloids) were treated with a low-dose regimen of UVA-1 phototherapy (starting dosage of 5 J/cm^2^, 3 sessions/week, with an increase of 5% per session, up to a treatment course of 25 sessions, reaching a maximum dose of 16.13 J/cm^2^) (Figure 1).

**Figure 2:**
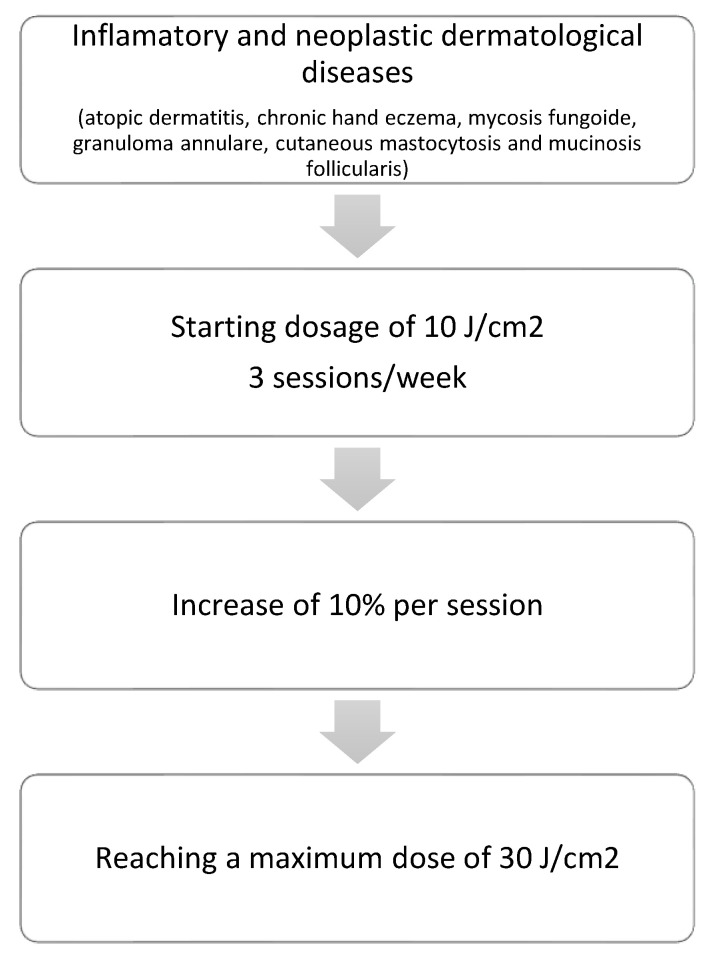
Treatment regimens for inflammatory and neoplastic dermatological diseases.

Patients with the other diseases (atopic dermatitis, chronic hand eczema, mycosis fungoides, granuloma annulare, cutaneous mastocytosis, and mucinosis follicularis) were prescribed a phototherapy scheme at slightly higher doses based on the literature available to date and the possibilities offered by our equipment (starting dosage of 10 J/cm^2^, 3 sessions/week, with an increase of 10% per session, up to a treatment course of 25 sessions, achieving a maximum dose of 30 J/cm^2^) (Figure 2).

In general, patients received intermittent therapies. Considering the severity of the disease in some patients at the time of treatment and assessing the appropriateness of prescribing maintenance therapy (mycosis fungoides, mastocytosis, chronic hand eczema, morphea, and advanced scleroderma), it was necessary to change the number of sessions per week (from 2 to 5 weekly) and the total of sessions of a treatment course (from 25 to 60 per cycle of treatment) in a few patients.

Therapy was delivered in a Waldmann UVA 302L phototherapy unit. Patients underwent UVA1 as monotherapy (systemic therapies were either not indicated or had previously failed in patients for whom they could be considered). Adjuvant treatment with moisturizers and topical corticosteroids was continued in those patients for whom we considered that it could be beneficial.

Patients who reached a 95% response rate in the scales performed were considered as complete responders. Likewise, patients with a response of at least 50% on the scales were considered as partial responders. Non-responders did not achieve these objectives. 

This study was conducted in accordance with the Helsinki Declaration of 1964 and all subsequent amendments and was approved by the Ethics Committee of Hospital Universitario San Cecilio (DER-HUSC-010) on October 2022.

## 3. Results

A total of 78 patients were treated with UVA1, of whom 46 patients (59%) were over 18 years of age, completed treatment, and gave their consent. We excluded 3 underage patients (4%) and 7 patients (9%) who did not sign the informed consent. During the study period, the COVID-19 pandemic occurred, and 22 patients (28%) were lost to follow-up and did not complete treatment.

Of the 46 patients, 36 were women (78%) and 10 were men (22%). Patient age ranged from 18 to 81 years (median 55 years). Fitzpatrick skin phototypes were documented in all analyzed patients: 0 patients were type I (0%), 19 patients were type II (41%), 25 patients were type III (54%), 2 patients were type IV (5%), 0 patients were type V (0%), and 0 patients were type VI (0%).

Among the 46 patients, those with sclerosing skin disorders predominated (30 patients). Thirteen patients in the study had morphea, there were eight with systemic sclerosis, five with keloids, two with extragenital lichen sclerosus, and two with scleredema. The remaining 16 patients had other skin disorders (Table 1). 

**Figure 3:**
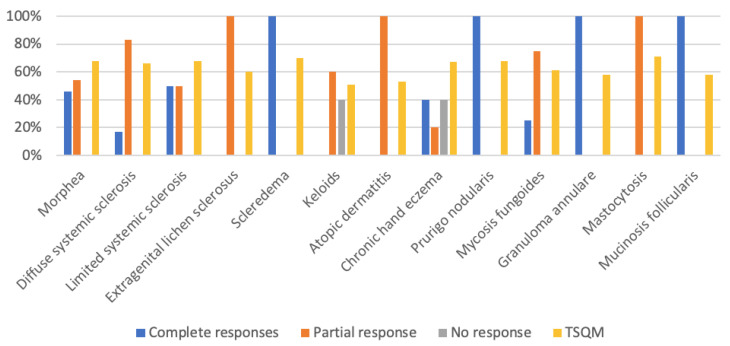
Skin diseases treated with UVA1 and clinical response (%).

The overall objective clinical response rate was 91.30% (42/46), obtaining complete response in 17 (36.96%) patients, partial response in 25 (54.34%), and no response in 4 (8.70%). High complete response rates were recorded in morphea, scleredema, or chronic hand eczema. In terms of the level of satisfaction objectively measured by TSQM-9 version 1.4, the average score was above 50 points in all of the entities treated, with particularly high scores obtained in mastocytosis, systemic sclerosis, morphea, scleredema, chronic hand eczema, or prurigo nodularis (over 65 points) (Figure 3).

## 4. Discussion

Phototherapy is a treatment used for multiple dermatological conditions. Numerous cells, including T and B lymphocytes, fibroblasts, dendritic cells, and immature mast cells, are affected by UVA1’s physiologic activities [1]. It is believed that UVA1’s therapeutic effectiveness for the treatment of T cell-mediated inflammatory and neoplastic skin illnesses, such as atopic dermatitis and mycosis fungoides, is due to its capacity to induce the apoptosis of invading T cells [2,3]. Proinflammatory cytokines, such as IL-12 and tumor necrosis factor (TNF)-alpha, as well as transforming growth factor (TGF)-beta, have been demonstrated to be suppressed by UVA1 radiation in human skin [5]. Interferon-gamma and ICAM-1, which are involved in lymphocyte activation and trafficking, are also decreased by UVA1. Matrix metalloproteinases, such as collagenase-1 generated by dermal fibroblasts, are enhanced by singlet oxygen species and hydrogen peroxide created because of UVA1 exposure [6]. The resulting increased collagen breakdown is thought to be the mechanism underlying the efficacy of UVA1 in the treatment of morphea and other sclerotic skin diseases [7]. Recently, Tognetti et al. [8] reported that UVA-1 phototherapy provides significant improvements in local tissue remodeling, rebalancing the alteration between profibrotic and antifibrotic pathways, and that these changes can be well monitored by high-frequency ultrasound.

The optimal therapeutic regimens and dosing of UVA1 are a matter of debate [4]. The doses used are highly variable among published studies and there are no evidence-based protocols that tell us about the dosage, duration, or the role of maintenance therapy [4]. There is some evidence that long courses at low doses are likely to be as effective as short courses at high doses [2]. The limitation of the studies is the fact that they were carried out in treatment courses limited to 12 weeks, not allowing enough time to assess changes in major immune processes and probably underestimating the potential effectiveness of this treatment in long courses [2]. UVA1 phototherapy has been developed mainly in the treatment of sclerosing diseases, being considered the first-line treatment in morphea [9,10].

In these diseases, it has potential efficacy due to its effect on immune dysregulation and collagen metabolism, which can soften lesions, reduce inflammation, and decrease dermal thickness. This has led to its development in morphea, scleroderma, and lichen sclerosus as main entities, among others [9]. 

In morphea, UVA1 phototherapy is considered one of the treatments of choice, with a level of evidence of efficacy similar to that of methotrexate [10]. Patients who could benefit would be those who show signs of clinical activity, as neither purely sclerotic nor atrophic plaques will respond [10]. The first study was published by Stege et al. [11], who found reduced thickness and increased elasticity in all 17 patients treated with UVA1 at high doses (130 J/cm^2^). Subsequently, Tuchinda C et al. [3] demonstrated greater efficacy of medium- and high-dose treatment compared with low-dose treatment in a study of 37 patients. Prasad et al. [1] concluded that the most evidence-based regimen for the treatment of morphea would be medium-dose regimens (60 J/cm^2^), 3–5 days per week for a total of 40 sessions. From the most recent studies, Furuhashi T et al. [12] published a series of three patients with morphea who responded satisfactorily to UVA1 phototherapy at a dose of 60 J/cm^2^, this fact was demonstrated by a histological study and elastography. In our group, six patients had a complete clinical response and seven had a partial response with a low-dose regimen. We also recorded good results in terms of patient satisfaction. Among patients with complete response, one had linear morphea; this variant will require longer treatment cycles [2]. In this case, we applied a total of 60 sessions. Our experience allows us to support that phototherapy with low-dose UVA 1 is a well-tolerated option with satisfactory results in these patients.

Regarding systemic sclerosis, the main studies carried out have focused on the treatment of hands [13]. It is known, from other studies on systemic sclerosis, that hands improve more slowly than other affected body parts, so this fact would be a limiting factor when evaluating the improvement of this disease in treatment with UVA1 among the literature [13]. Connoly et al. [10] evaluated the response to treatment with low (20–40 J/cm^2^), medium (>40–80 J/cm^2^), and high (>80–120 J/cm^2^) doses of UVA1 in 16 patients with systemic sclerosis and concluded that the effect of UVA1 treatment in sclerosing diseases is dose-dependent, showing a better response with high doses than with medium or low doses. Similarly, El-Mofty et al. [14] studied the response to treatment with low-dose UVA1 (5–20 J/cm^2^) in 15 patients with systemic sclerosis (10 with limited systemic sclerosis and 5 patients with diffuse systemic sclerosis) and reported a poor therapeutic outcome. Durand et al. [15] demonstrated a decrease in skin thickening and an improvement in functional discomfort in hands exposed to UVA treatment (40 J/cm^2^), but the result was not significantly different from that observed in the control group. The low-dose regimen did not influence the flexion or extension function of the hands.

**Figure 4:**
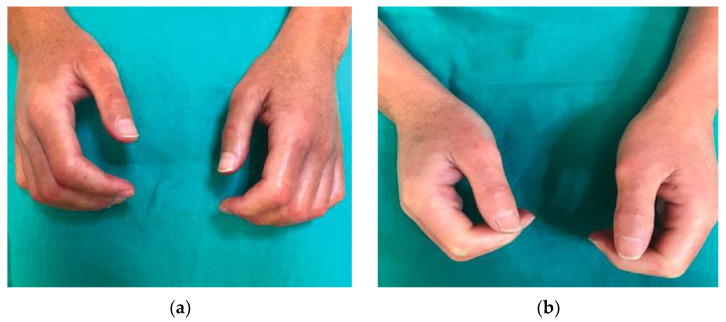
Patient with systemic sclerosis limited to the hands: (**a**) Patient’s condition prior to treatment with UVA1 phototherapy; (**b**) response of the patient after treatment with 25 sessions of UVA1 phototherapy at low doses.

Nevertheless, there are several publications that defend the efficacy of low-dose UVA1 phototherapy in the treatment of systemic sclerosis. Kreuter et al. [16] observed significant skin softening and improved finger mobility in 16 of 18 patients who received low doses (30 J/cm^2^) of UVA1 phototherapy on the hands. This therapeutic response was confirmed by ultrasound measurements, which showed a significant increase in skin elasticity in 15 patients and a reduction in dermal thickness in 14 patients. Similarly, Rose et al. [17] observed another improvement in patients who did not respond to previous therapies with low-dose (15–40 J/cm^2^) UVA regimens. Pitney et al. [2] reported only a partial response in the four patients with systemic sclerosis included in their research who received UVA1 treatment at low doses (30 J/cm^2^). Our results, with objective and perceived improvementd in all patients treated (8/8), propose that benefits could be obtained by performing cycles of treatment with lower cumulative doses (Figure 4).

Regarding extragenital lichen sclerosus, Kreuter et al. [18] published the largest study that involved the treatment of 10 patients with 40 sessions of ultraviolet A1 (UVA1) light at a dose of 20 J/cm^2^ four times per week. All patients exhibited lesion softening and improvement in pigmentation. In our group, two patients showed a partial response. Both had been refractory to treatment with topical corticosteroids. Our results are in line with those previously published, suggesting that low-dose UVA1 could be an effective treatment in patients suffering from extragenital lichen sclerosus.

**Figure 5:**
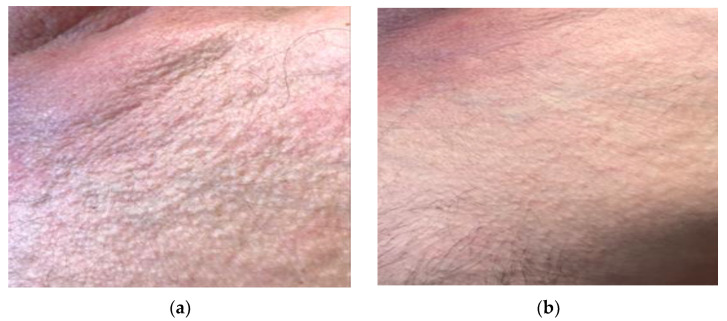
Patient with scleredema and orange skin appearance: (**a**) Patient’s condition prior to treatment with UVA1 phototherapy; (**b**) complete response of the patient after treatment with 25 sessions of UVA1 phototherapy at low doses.

Scleredema clinically presents as widespread diffuse, woody induration of the skin. Support for the use of UVA1 in scleredema can be derived from observations in a few patients who demonstrated moderate to marked improvement during UVA1 therapy [19,20,21,22,23]. Treatment is usually administered two to five times per week. The optimal treatment regimen remains unclear. Our three patients would be the largest series published to date with a low-dose schedule. The results suggest that it can be considered as an effective therapeutic option, although further studies are needed to confirm this hypothesis (Figure 5).

**Figure 6:**
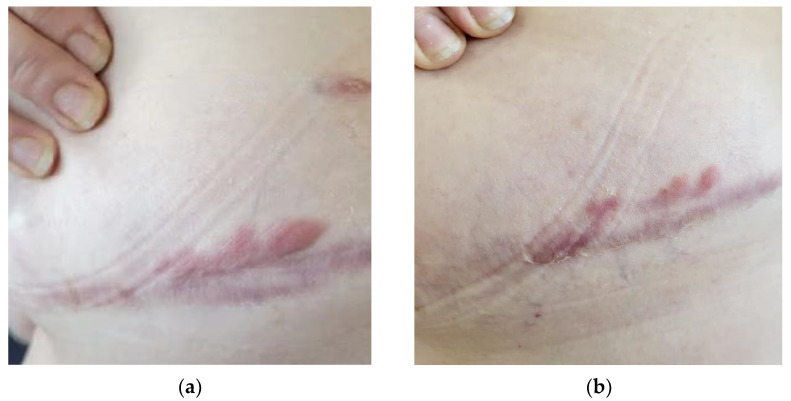
Patient with submammary keloids on the right chest: (**a**) Patient’s condition prior to treatment with UVA1 phototherapy; (**b**) reduction in the erythema of the lesion was observed after 25 sessions. In addition, there was a remarkable reduction in pain and discomfort reported by the patient.

Furthermore, UVA1 phototherapy has been suggested to be effective for the treatment of keloids, although the literature on this topic is still scarce [24]. The efficacy of UVA1 is attributed to the induction of collagenase I production by fibroblasts and decreased synthesis of procollagen [25]. The reported histological examination of keloids after UVA-1 revealed the normalization of collagen and elastic fibers’ distributions, the reduction in mucin and cellularity, and the increase in vascular structures. Clinical and symptomatological improvement has also been referred to in other publications as an important benefit, with reductions in itching, discomfort, and tenderness [25]. Our series of patients with keloids treated with low-dose UVA1 is one of the largest. This may be considered a safe and effective alternative in keloid therapy, particularly in cases of significant extension or associated with important symptomatology, a main point in keloid therapy (Figure 6).

Regarding severe atopic dermatitis, the role of UVA1 phototherapy in treatment is well established [26,27,28,29]. A systematic review [30] of nine randomized trials found that, in adult patients with acute, severe atopic dermatitis, phototherapy with UVA1 was faster than that with conventional UVA/UVB in inducing clinical improvement, with a peak response after 10 treatments. In studies of patients with atopic dermatitis, a medium UVA1 dose (50 J/cm^2^) was superior to a low-dose UVA1 regimen (10 J/cm^2^), but no significant difference was noted between the medium- and high-dose regimens. Moreover, low-dose UVA1 (30 J/cm^2^) was less effective than UVA/UVB therapy, whereas high-dose UVA1 therapy (130 J/cm^2^) was superior to UVA/UVB phototherapy [26]. Malinowska K et al. [31] reported that medium-dose UVA1 phototherapy had significant antipruritic effects, improved atopic dermatitis (as measured by the SCORAD index), and improved quality of life (as measured by the DLQI questionnaire). Our results support the usefulness of UVA1 phototherapy at low doses in atopic dermatitis, which could be positioned as a fast and effective alternative in the treatment of this field. A cumulative dose administered in daily high-dose treatments has not yet been compared with a longer course of daily low doses equivalent to the same cumulative dose. As the equipment needed to administer high doses is bulky and expensive compared with low-potency equipment, it would be useful to compare these regimens.

Regarding mycosis fungoides, several published studies suggest the efficacy and safety of UVA1 phototherapy for the treatment of cutaneous T-cell lymphoma, particularly mycosis fungoides [32]. The fact that malignant CD4^+^ T cells appear to be more sensitive to UVA1 radiation-induced apoptosis than normal CD4^+^ T cells supports the convenience of this treatment in the management of cutaneous T-cell lymphomas [32]. Patients with stage IA-IIA are candidates for treatment with phototherapy in monotherapy [33]. In patients who have been refractory to monotherapy or who are in stage IIB or higher, combination therapy with systemic agents should be proposed [32,33]. In 2013, in a study with 14 patients [34], no differences in effectiveness were demonstrated between low (20 J/cm^2^), medium (65 J/cm2), or high (100 J/cm^2^) doses. The most-studied low-dose regimen has been 30 J/cm^2^ at 5 days a week for 5 weeks. In our series, in which we treated patients with mycosis fungoides with plaque lesions (commonly refractory to UVB-BE phototherapy), we observed improvement in all of them (one of whom had a complete response). Our data, in accordance with what has been previously published, are consistent with UVA1 in the treatment of mycosis fungoides, especially in its clinical presentation in plaques.

With respect to granuloma annulare, although UVA1 appears to have some efficacy for generalized granuloma annulare, further studies are necessary to identify the optimal treatment regimen [35,36,37]. In fact, the latest review on this subject by Mukovozov et al. [38] positions it as an alternative to treatment with narrowband UVB phototherapy, which is considered as the first-line phototherapy for this entity. Our results support the hypothesis already put forward by other published studies that UVA1 is possibly effective for generalized granuloma annulare.

UVA1 therapy has also been demonstrated to improve the symptomatology of cutaneous mastocytosis [39]. Stege et al. [40] reported improvement of symptoms in 4/4 patients after 2 weeks of high-dose therapy (130 J/cm^2^). Improvement of symptoms was maintained at the 10-month follow-up and continued for at least 23 months. All the cases published so far used UVA1 at high doses [39,40]. We present the first case of a good response to UVA1 at low doses. More experience will be needed to determine the optimal treatment regimen.

**Figure 7:**
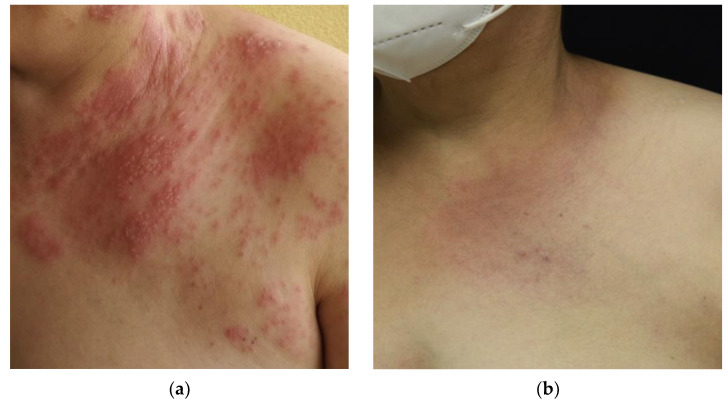
Patient with mucinosis follicularis: (**a**) Patient’s condition prior to treatment with UVA1 phototherapy after 30 sessions; (**b**) complete response of the patient after treatment with UVA1 at low doses.

Regarding mucinosis follicularis, von Kobyletzki et al. [41] suggested that medium-dose UVA1 phototherapy may be highly effective in the treatment of resistant mucinosis follicularis, even in patients with progressive disease. In our series, we observed a complete response after treatment in the only patient treated, supporting this hypothesis and providing the first case of a complete response to UVA1 phototherapy at low doses (Figure 7).

Although prurigo nodularis is a therapeutical challenge, phototherapy has been described as an effective tool in the management of the disease, with satisfactory results in most cases [42]. S. Rombold et al. [43] described a satisfactory response with medium doses of UVA1 in 14 out of 17 patients with PN. Likewise, Bruni et al. [44] demonstrated clinical improvement in 15 out of 19 patients with prurigo nodularis after cycles of low-dose UVA1. In our group, the two patients treated had total improvement in their clinical condition.

For chronic recurrent hand eczema, UVA1 phototherapy can be used with great efficacy [45]. UVA1 phototherapy has been shown to improve subjective and objective complaints in dyshidrotic eczema [46]. In this study, 28 patients were randomly assigned to a treatment group (UVA1 phototherapy (40 J/cm^2^ at 5 times per week for 3 weeks)) and a placebo group. As well as scaling, signs of skin inflammation were also significantly improved with irradiation compared with the placebo. In addition, a controlled study [47] showed that there was no difference in the efficacy of PUVA cream therapy compared with UVA1 phototherapy at high doses (130 J/cm^2^). UVA1 phototherapy has the practical advantage of eliminating the need to apply a cream to the hands and feet before irradiation and the need for constant light protection after irradiation. Another study [48] compared the therapeutic effect of UVA1 phototherapy at high and low doses in 27 patients (9 men and 18 women) with dyshidrotic eczema of the hands (*n* = 18) and feet (*n* = 9). The cumulative light dose was 730 J/cm^2^ in the low-dose range and 1720 J/cm^2^ in the high-dose range, with irradiation performed five times per week for 3 weeks. In each case, the foot or hand was irradiated with a low dose of UVA1 (50 J/cm^2^). In comparison, the other corresponding affected body part was treated with a high dose of UVA1 (130 J/cm^2^). The results showed no difference in efficacy between low- and high-dose UVA1 treatment after therapy. These studies demonstrate that both high-dose UVA1 phototherapy (maximum dose of 130 J/cm^2^) and lower-dose phototherapy (maximum dose of 50 J/cm^2^) can be used effectively in the treatment of dyshidrotic eczema of the hands and feet. In summary, selective UVA1 phototherapy at low doses represents a good and potent alternative to previously established forms of treatment for dyshidrotic hand and foot eczema. Data comparing the therapeutic efficacy of low- and high-dose UVA1 phototherapy in chronic hyperkeratotic eczema are not yet available. In our patients, the results using low doses of UVA1 were variable, with two patients showing a complete response, one a partial response, and two showing no response at all. Further studies are needed to reach a consensus on the optimal treatment.

Regarding the level of satisfaction, we found a relationship between the degree of satisfaction and the therapeutic response, except in granuloma annulare, where, despite good therapeutic results, the degree of satisfaction was not high. Furthermore, more severe patients showed more limited clinical improvements and lower levels of satisfaction, such as patients with keloids.

All patients tolerated UVA1 well. We did not report any serious adverse effects, the most frequent being skin dryness or tanning. The main inconvenience reported by the patients was the difficulty in reconciling treatment with other activities, such as work schedules. Potential side effects, such as photodermatoses or drug-induced photosensitivity, were not experienced. This study has some limitations. Firstly, the number of patients for most diseases was small. Furthermore, it was a retrospective study, so there was a loss of patients during follow-up that reduced the potential sample size. In addition, we should take into account the existence of a certain subjectivity when measuring improvement in some of the scales used, especially in sclerosing disorders, where we did not have more objective measurement techniques. There is also the difficulty in defining the therapeutic endpoints and the duration of maintenance therapy in chronic disorders.

## 5. Conclusions

We present a review of treatment with UVA1 phototherapy at low doses with good response in a wide variety of dermatological pathologies, many of which have few related publications. Our results highlight the benefit of cumulative, rather than individual, dosing in achieving patient outcomes. Our study also provides, with respect to a previously published low-dose series, a record of responses using disease-specific rating scales and a record of the degree of patient satisfaction with treatment using the TSQM-9 version 1.4 scale. We suggest that UVA1 phototherapy at low doses is a safe and effective treatment for skin diseases, mainly in scleroderma, morphea, and atopic dermatitis. For this reason, with the advance in the knowledge of this modality, treatment protocols should be established for each entity.

## Figures and Tables

**Table 1:** Skin diseases treated with UVA1, clinical response, and level of satisfaction.

Diagnosis	Patients	Sex (Male/Female)	Median Age (Range)	Range Sessions per Cycle	Median Total Joules (Range)	Response Complete (>95%)	Response Partial (>50%)	No Response	Median TSQM (Range)	Adverse Effects
Morphea	13	2/11	56 (18–76)	17–60	321 (111–632)	6 (46.15%)	7 (53.85%)	0 (0%)	68 (41–72)	Two patients tanning
Diffuse systemic sclerosis	6	0/6	57 (48–81)	10–35	449 (214–1529)	1 (16.67%)	5 (83.33%	0 (0%)	65,5 (60–72)	Not referred
Limited systemic sclerosis	2	0/2	57.5 (42–73)	20–35	377 (323–431)	1 (50%)	1 (50%)	0 (0%)	68 (66–71)	No referred
Extragenital lichen sclerosus	2	0/2	69 (62–76)	30	470 (330–610)	0 (0%)	2 (100%)	0 (0%)	60.5 (56–65)	One patient tanning
Scleredema	2	1/1	59.5 (55–64)	25–30	619 (108–1130)	2 (100%)	0 (0%)	0 (0%)	70.5 (69–72)	No referred
Keloids	5	2/3	36 (25–57)	10–30	331 (47–714)	0 (0%)	3 (60%)	2 (40%)	51 (41–67)	No referred
Atopic dermatitis	2	0/2	36.5 (23–50)	20–25	333 (229–437)	0 (0%)	2 (100%)	0 (0%)	53 (45–61)	One patient skin xerosis
Chronic hand eczema	5	2/3	54 (23–69)	30	753 (513–805)	2 (40%)	1 (20%)	2 (40%)	67 (41–72)	No referred
Prurigo nodularis	1	0/1	67	25	554	1 (100%)	0 (0%)	0 (0%)	68	No referred
Mycosis fungoides	4	3/1	49 (24–51)	30–40	853 (567–1679)	1 (25%)	3 (75%)	0 (0%)	61.5 (48–71)	One patient skin xerosis and transient erythema
Granuloma annulare	2	0/2	64.5 (57–72)	20–25	158 (118–198)	2 (100%)	0 (0%)	0 (0%)	58 (51–65)	No referred
Mastocytosis	1	0/1	40	30	1686	0 (0%)	1 (100%)	0 (0%)	71	No referred
Mucinosis follicularis	1	0/1	59	30	510	1 (100%)	0 (0%)	0 (0%)	58	No referred
TOTAL	46	10/36	55 (18–81)			17 (36.96%)	25 (54.34%)	4 (8.70%)	65 (41–72)	

## Data Availability

Not applicable.
